# Immediate Platelet Inhibition Strategy for Comatose Out-of-Hospital Cardiac Arrest Survivors Undergoing Percutaneous Coronary Intervention and Mild Therapeutic Hypothermia

**DOI:** 10.3390/jcm13072121

**Published:** 2024-04-06

**Authors:** Peter Kordis, Jernej Berden, Ursa Mikuz, Marko Noc

**Affiliations:** 1 Center for Intensive Internal Medicine, University Medical Center Ljubljana, 1000 Ljubljana, Slovenia; peter.kordis@kclj.si (P.K.);; 2 Faculty of Medicine, University of Ljubljana, 1000 Ljubljana, Slovenia

**Keywords:** cangrelor, cardiac arrest, platelet inhibition, percutaneous coronary intervention, stent thrombosis

## Abstract

**Background:** Comatose survivors of out-of-hospital cardiac arrest (OHCA) undergoing percutaneous coronary intervention (PCI) and target temperature management (TTM) are at increased risk of stent thrombosis (ST), partly due to delayed platelet inhibition even with more potent P2Y_12_ agents. We hypothesized that periprocedural cangrelor would induce immediate platelet inhibition, bridging the “P2Y_12_ inhibition gap”. **Methods:** In our pilot study, we randomized 30 comatose OHCA patients undergoing PCI and TTM (32–34 °C) into cangrelor and control groups. Both groups received unfractioned heparin, acetylsalicylic acid, and ticagrelor via enteral tube. The cangrelor group also received an intravenous bolus of cangrelor followed by a 4 h infusion. Platelet inhibition was measured using VerifyNow^®^ and Multiplate^®^ ADP at baseline and 1, 3, 5, and 8 h post PCI. **Results:** Patient characteristics did not differ between groups. VerifyNow^®^ showed significantly decreased platelet reactivity with cangrelor at 1 h (30 vs. 221 PRU; *p* < 0.001) and 3 h (24 vs. 180 PRU; *p* < 0.001), with differences at 5 and 8 h. Similarly, the proportion of patients with high on-treatment platelet reactivity (HPR) in the cangrelor group was significantly lower at 1 h (0% vs. 67%; *p* < 0.001) and 3 h (0% vs. 47%; *p* = 0.007). Multiplate^®^ ADP was also decreased at 1 h (14 vs. 48 U; *p* < 0.001) and 3 h (11 vs. 42 U; *p* = 0.001), with no difference at 5 and 8 h. The occurrence of bleeding events was similar in both groups. **Conclusions:** Cangrelor safely induced immediate and profound platelet inhibition. We observed no significant drug–drug interaction with ticagrelor.

## 1. Introduction

Patients who remain comatose after out-of-hospital cardiac arrest (OHCA) present unique challenges in medical care after percutaneous coronary intervention (PCI) compared to conscious patients. They require mechanical ventilation and are unable to orally ingest antithrombotic drugs. In addition to intravenous unfractionated heparin and acetylsalicylic acid, all patients receive antiplatelet therapy with a P2Y_12_ receptor inhibitor, traditionally administered via enteral tube, potentially causing a delay in action [1]. Moreover, the efficacy of antiplatelet drugs is influenced by numerous other factors: delayed absorption due to gastroparesis and gastrointestinal hypoperfusion after OHCA, therapeutic hypothermia, and increased platelet reactivity in post-resuscitation syndrome [2,3]. The delayed onset of action of enteral P2Y_12_ receptor inhibitors can also be attributed to the use of morphine and synthetic opioids in early post-resuscitation care, which impact gastric motility [4]. These mechanisms render comatose OHCA patients more prone to acute and subacute stent thrombosis (ST) [5]. Furthermore, frequent post-resuscitation chest injuries and respiratory tract damage due to intubation present higher risks of bleeding [6].

Stent thrombosis is more common in comatose OHCA patients compared to patients without cardiac arrest [7]. Retrospective data suggest the incidence ranges from 2.7% to 31%, whereas in patients with acute myocardial infarction without cardiac arrest, it is approximately 1% [8,9,10,11,12]. The findings of the first study examining definite ST in OHCA patients post PCI, confirmed through systematic coronary angiography or autopsies, revealed ST in 19% of cases, with one-fifth of them being clinically silent [13]. Among cases where the timing of events was known, 57% occurred within the first day of hospitalization [13]. Optimizing anticoagulant and antiplatelet therapy in comatose OHCA patients is crucial for limiting the incidence of ST, a severe complication linked to high mortality rates. However, it is important to note that, so far, insufficient platelet inhibition in OHCA patients has not been causally linked to adverse thrombotic events, as multiple factors can influence stent thrombosis.

In mild hypothermia up to 33 °C, primary and secondary hemostasis are affected, with increased platelet function and inhibition of coagulation most commonly described [14]. However, the utilization of mild therapeutic hypothermia in a large TTM study was not associated with increased risk of bleeding [15]. In nearly one-third of OHCA patients undergoing therapeutic hypothermia, insufficient platelet inhibition was reported even after 24 h, despite the use of more potent P2Y_12_ inhibitors [16]. A randomized study conducted by Steblovnik et al. demonstrated that adequate platelet inhibition with a P2Y_12_ receptor inhibitor in comatose OHCA patients was achieved only three to four hours after ticagrelor administration; the gap was even longer when using less potent clopidogrel [5]. This period of inadequate platelet inhibition may be one of the factors predisposing patients to platelet aggregation on the stent and subsequent ST, a phenomenon most commonly observed within the first day post PCI [13]. 

To enhance the efficacy of the second antiplatelet medication, intravenous antiplatelets can serve as a bridging option to achieve sufficient platelet inhibition. While glycoprotein IIb/IIIa inhibitors (GPIs) are highly potent, they may lead to significant adverse effects due to their sustained impact on platelet function even after discontinuation [17]. Current guidelines recommend GPI use only in cases of suboptimal angiographic results, such as slow reflow or no reflow phenomena [1]. Cangrelor, the only intravenous P2Y_12_ inhibitor, is an active agent that reversibly inhibits platelet aggregation with a rapid onset of action and a short half-life of a few minutes [18,19]. Its intravenous administration bypasses issues related to delayed absorption, systemic inflammatory response, or other shock states, making it more effective than oral P2Y_12_ inhibitors without increased bleeding risk [2]. Platelet function returns to normal within one hour following cessation of the infusion [20]. In the CHAMPION meta-analysis, researchers compared ischemic and bleeding events among patients without OHCA requiring elective or urgent PCI. They discovered that the efficacy of GPI and cangrelor was similar; however, the cangrelor group experienced significantly fewer bleeding events [21]. Prueller et al. conducted a retrospective study comparing cangrelor infusion followed by a switch to an oral P2Y_12_ inhibitor with the use of an oral P2Y_12_ inhibitor alone in OHCA patients. Their findings confirmed a more significant antiplatelet effect with cangrelor without an increase in bleeding frequency [2]. Another retrospective study in a smaller cohort of OHCA patients examined the addition of cangrelor to standard therapy, including early administration of ticagrelor via an enteral tube. Significant differences in platelet inhibition were noted as early as one hour after the loading dose and the start of a four-hour cangrelor infusion. In the control group, only 1 out of 9 patients achieved adequate inhibition one hour post PCI, whereas in the cangrelor group, 11 out of 13 patients achieved this outcome. This effect persisted until the end of the infusion, with no notable differences in the incidence of major bleeding or documented ST [22]. The results of this and the CANTIC study showed that there are no interactions between cangrelor and ticagrelor when both drugs are used simultaneously [22,23]. To our knowledge, there are no studies prospectively comparing the effects of P2Y_12_ inhibitors following standard clinical practice with those receiving an additional cangrelor infusion to bridge the period of inadequate inhibition.

We hypothesized that administering a bolus and a four-hour infusion of cangrelor at the onset of PCI in comatose OHCA patients would induce immediate platelet inhibition and bridge the gap until a sufficient antiplatelet effect is achieved through crushed ticagrelor tablets administered via an enteral tube. Limited late-breaking data from our study have been previously published as a research correspondence letter [24].

## 2. Materials and Methods

### 2.1. Study Setting and Patients

Our randomized controlled pilot trial (NCT04005729) enrolled consecutive comatose survivors of OHCA with presumed cardiac etiology admitted to the University Medical Center Ljubljana (Ljubljana, Slovenia). Included patients underwent immediate PCI with stenting and targeted temperature management (TTM) with therapeutic hypothermia of 32–34 °C. All patients were intubated and mechanically ventilated, and intensive care procedures followed a standardized post-resuscitation protocol, including parenteral opioid use [25]. TTM aimed at 32–34 °C was initiated upon admission and maintained for 24 h, followed by gradual rewarming [26]. The study protocol was approved by the National Ethical Committee (KME 0120-61/2019/8) and Agency for Medicinal Products and Medical Devices of the Republic of Slovenia (JAZMP 1050-58/2019-3).

Following coronary angiography and prior to planned PCI, patients were randomly allocated in a 1:1 ratio to either the cangrelor or the control group using a random table and a sealed envelope system. The main exclusion criteria were age over 70 years, requirement for venoarterial extracorporeal circulation (VA-ECMO), active bleeding upon arrival to the catheterization laboratory, history of ischemic stroke, current use of P2Y_12_ inhibitor or anticoagulation therapy, and intraprocedural administration of GPI. All patients received an intravenous bolus of 250 to 500 mg acetylsalicylic acid (Aspegic^®^, Sanofi-Aventis, Paris, France) and unfractionated heparin (UFH) to achieve an activated clotting time (ACT) of 250 to 300 s. At the start of PCI, the cangrelor group received an intraprocedural intravenous bolus of cangrelor (30 mcg/kg), immediately followed by a 4 h infusion (4 mcg/kg/min). Patients assigned to the control group received no intervention. A nasogastric tube was inserted as soon as possible upon admission to the intensive care unit and a loading dose of 180 mg ticagrelor (Brilique^®^, AstraZeneca, Cambridge, UK) was administered as crushed and dissolved tablets in both groups.

### 2.2. Platelet Reactivity

Upon arrival at the catheterization laboratory, blood samples were taken to determine ACT, complete blood count values, and baseline platelet reactivity using aggregometry. Blood samples for platelet inhibition assessment were collected at 1, 3, 5, and 8 h from randomization, corresponding to the start of PCI or the initiation of cangrelor infusion in the cangrelor group. Baseline platelet reactivity and the degree of platelet inhibition were determined using the VerifyNow^®^ System (Accriva Diagnostics, Piscataway, NJ, USA) and Multiplate^®^ (Roche, Basel, Switzerland) aggregometry methods. The VerifyNow^®^ bedside analyzer provides rapid functional determination of platelet reactivity using optical aggregometry. The analyzer detects changes in optical density after platelet aggregation and numerically expresses it as PRUs (platelet reactivity units). In the case of a P2Y_12_ inhibitor effect, platelet aggregation is reduced, resulting in lower PRU values. Potential high on-treatment platelet reactivity (HPR) was defined as PRU values above 208, according to the literature [27]. Multiplate^®^ test is based on impedance aggregometry, measuring changes in electrical conductivity. Reduced conductivity between the electrodes in platelet aggregation is expressed by the device as values in aggregation units (AUs). The effectiveness of P2Y_12_ inhibitors is reflected in reduced platelet aggregation and lower AU values. High on-treatment platelet reactivity was defined as AU values above 46, based on recommendations [27].

### 2.3. Primary Endpoints

The primary efficacy endpoint was the degree of platelet inhibition in the cangrelor and control groups at 1, 3, 5, and 8 h after passage of the PCI guidewire, as measured by the VerifyNow^®^ and Multiplate^®^ tests, following established protocols. High on-treatment platelet reactivity was defined as PRU > 208 and AU > 46. The primary safety endpoint included bleeding events, defined as Bleeding Academic Research Consortium (BARC) score type 2, 3, or 5, or the necessity to discontinue cangrelor infusion, as determined by the treating intensivist [28].

### 2.4. Secondary Endpoints

The secondary outcome measures in the study included:

- Angiographic result—Final Thrombolysis in Myocardial Infarction (TIMI) Flow. Final angiographic result at the end of the procedure was assessed by an independent blinded interventional cardiologist;
- Rate of stent thrombosis. Defined as definite or probable ST based on the Academic Research Consortium (ARC) classification during the index patient hospitalization. Definite ST was confirmed at angiography or autopsy, while probable stent thrombosis included unexplained death within 30 days after PCI or new myocardial infarction in the PCI vessel territory;
- Timing of ST. Stent thrombosis events were categorized as acute (within 24 h after stenting) or subacute (24 h to 30 days after stenting);
- Survival. We described survival to discharge from the hospital during the index hospitalization, up to 90 days;
- Survival with favorable neurological outcome—Cerebral Performance Category (CPC). We assessed survival to discharge from the hospital based on CPC scores, with CPC 1–2 indicating a favorable neurological outcome. Evaluation was conducted during the index hospitalization, up to 90 days.

### 2.5. Power Analysis and Statistical Analysis

Based on the only published retrospective non-randomized study comparing the effect of standard ticagrelor treatment with a group receiving the addition of cangrelor (bolus and four-hour infusion), the outcomes showed that after one hour, 11 out of 13 patients in the cangrelor group had sufficient platelet inhibition, compared to 1 out of 9 patients in the control group [22]. Setting the Type I error rate at 0.05 (α = 0.05) and study power at 0.9 (β = 0.1), a sample size of 14 patients was required to test the null hypothesis.

We performed the statistical analysis using IBM SPSS 17.0 (SPSS Inc., Chicago, IL, USA, IBM Company, 2008, Armonk, NY, USA). Numerical variables with a normal distribution were reported as the mean and standard deviation (SD), while other numerical variables were reported as the median and interquartile range. Categorical variables were reported as counts and percentages. For comparisons between groups, we used the independent samples *t*-test for normally distributed numerical variables, the Mann–Whitney U test for skewed numerical variables, and the Chi-square or Fisher’s exact test for categorical variables. A *p*-value of <0.05 was considered statistically significant.

## 3. Results

### 3.1. Baseline Demographics

**Figure 1:**
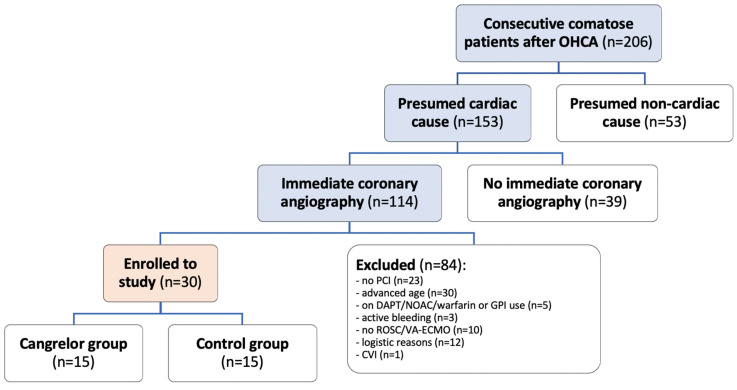
Study flowchart. OHCA—out-of-hospital cardiac arrest; PCI—percutaneous coronary intervention; DAPT—dual antiplatelet therapy; NOAC—non-vitamin K antagonist oral anticoagulants; GPI—glycoprotein IIb/IIIa inhibitors; ROSC—return of spontaneous circulation; VA-ECMO—venoarterial extracorporeal membrane oxygenation; CVI—cerebrovascular insult.

Among 153 consecutive comatose survivors of OHCA with presumed cardiac origin admitted to our hospital between July 2019 and November 2021, immediate coronary angiography was performed in 114 patients (75%). Of those, 84 patients met the exclusion criteria, predominantly due to factors such as advanced age (n = 30) or lack of PCI (n = 23); additional details are outlined in Figure 1. Thirty patients were enrolled in the study and randomized into the cangrelor and control groups in a 1:1 ratio.

No differences in gender, age, and cardiovascular risk factors were observed between the cangrelor and control groups; 90% of OHCA patients had no documented history of ischemic heart disease (Table 1). The circumstances of cardiac arrest and resuscitation did not differ between the groups. Following ROSC, ST segment elevation myocardial infarction (STEMI) was recorded in the 12-lead ECG in 70% of patients. Ischemic changes were observed in the remaining cases that did not meet the criteria for STEMI.

After admission to the ICU, there were no differences in the results of laboratory tests and the assessment of left ventricular ejection fraction (Table 2). Similarly, there were no differences in peak troponin levels recorded during hospitalization.

All patients were subjected to therapeutic hypothermia, with the majority (87%) receiving an opioid infusion as continuous analgesia on the day of admission. Cardiogenic shock was present in four (27%) patients in the cangrelor group, with one (7%) requiring mechanical support with an intra-aortic balloon pump (IABP). In the control group, cardiogenic shock was identified in seven (47%) patients, and seven patients required IABP support. There were no significant differences between groups in the prevalence of cardiogenic shock (*p* = 0.256) and the use of IABP (*p* = 0.080). One patient in the cangrelor group required venoarterial ECMO on the second day of hospitalization.

### 3.2. Angiographic Characteristics

Target coronary artery occlusion was observed in 22 patients (73%). Except for a higher prevalence of multivessel disease in the cangrelor group (73% vs. 27%, *p* = 0.011), no differences were noted between groups in other characteristics of diagnostic angiography and PCI strategy (Table 3). Bifurcation stenting was not performed in any patient. In all patients who underwent successful PCI, TIMI flow through the target coronary artery was adequate (TIMI 2–3).

### 3.3. Platelet Inhibition and Stent Thrombosis

Intravenous acetylsalicylic acid was administered to all patients before admission, with no significant difference in the average dose between the cangrelor and control groups (333 mg vs. 357 mg, *p* = 0.657). Similarly, the doses of UFH administered were comparable between the two groups. The total dose of UFH given before and during PCI was 120 IU/kg in the cangrelor group and 133 IU/kg in the control group (*p* = 0.419). Additionally, there were no differences in the interval from the start of PCI to the administration of ticagrelor via enteral tube, with 56 min in the cangrelor group and 64 min in the control group (*p* = 0.252).

**Figure 2:**
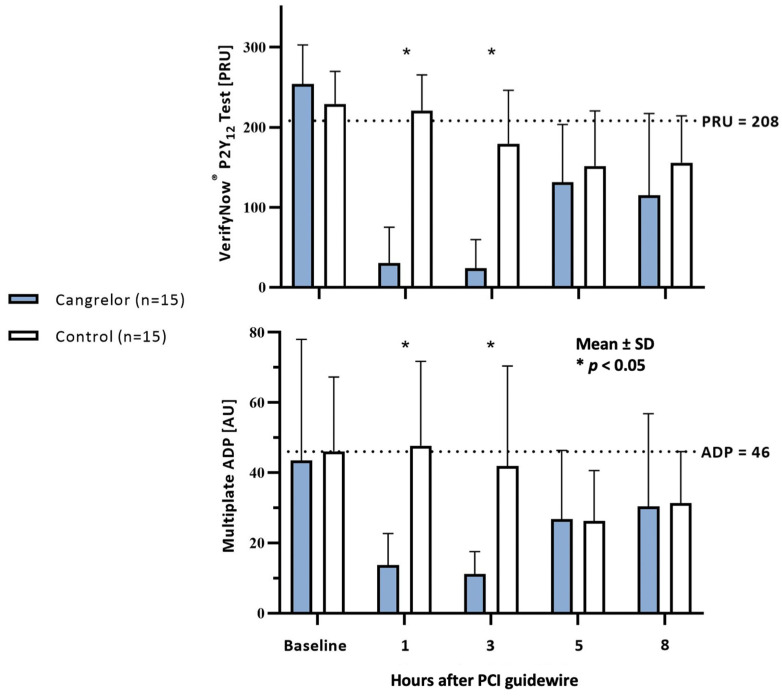
Platelet reactivity assessed by VerifyNow^®^ and Multiplate^®^ ADP assays in the cangrelor group (blue columns) and control group (white columns). Baseline status before percutaneous coronary intervention (PCI) and platelet reactivity at 1, 3, 5, and 8 h after PCI are shown. High platelet reactivity is indicated by dashed lines.

Baseline platelet reactivity, measured by the VerifyNow^®^ PRU test before PCI, did not differ between the groups. However, measurements at one hour (30 vs. 221 PRU, *p* < 0.001) and three hours (24 vs. 180 PRU, *p* < 0.001) after PCI revealed more pronounced platelet inhibition in the cangrelor group (Figure 2). Measurements taken at five and eight hours after PCI did not differ between the groups. Notably, at one and three hours post PCI, a higher proportion of patients in the control group showed HPR with PRU > 208, while the proportions before PCI and at five and eight hours post PCI were comparable (Table 4).

Platelet reactivity measurements with the Multiplate^®^ ADP test were comparable between groups before PCI, whereas platelet inhibition at one (14 vs. 48 AU, *p* < 0.001) and three hours (11 vs. 42 AU, *p* = 0.001) was more pronounced in the cangrelor group (Figure 2). Platelet inhibition at five and eight hours after PCI did not differ between groups. Additionally, a significantly higher proportion of patients with HPR (AU > 46) was observed at one and three hours after PCI in the control group (Table 5).

### 3.4. Secondary Endpoints

During the 30 day follow-up period, two cases of ST were confirmed in the cangrelor group and three in the control group (Table 6). All instances were definitive ST, with four confirmed through repeat coronary angiography and one during autopsy. The timing of ST was unknown in three cases (clinically silent ST), while in the remaining two cases, thrombosis occurred on the first and fourth day of hospitalization. The average doses of acetylsalicylic acid and cumulative UFH dose in patients with confirmed ST were 420 mg and 107 IU/kg body weight, respectively, with an average time from the start of PCI to the administration of oral P2Y_12_ inhibitor being 52 min. In one patient with ST, randomized to cangrelor group, multivessel PCI was performed during the initial coronary angiography.

During the administration of the cangrelor infusion, we did not detect any instances of HPR among patients in the cangrelor group. Nevertheless, one patient displayed HPR at five and eight hours following the cessation of the infusion, without documented ST.

There were no differences in the occurrence of bleeding events between groups. In the cangrelor group, three patients experienced bleeding, including two cases classified as BARC 1, which did not require intervention, presenting bleeding from the gums and hematuria after urinary catheter insertion. One BARC 2 event in cangrelor group involved recurrent bleeding from a scalp laceration following a fall during cardiac arrest. The infusion of cangrelor was discontinued, and the patient did not require transfusion or other interventions. In the control group, one patient experienced bleeding from the oral cavity and hematuria (classified as BARC 1). 

There was no difference in survival to hospital discharge between groups (67% vs. 60%, *p* = 0.705). All 19 surviving patients had a favorable neurological outcome at discharge, defined as CPC 1 or CPC 2. Among the majority (73%) of deceased patients, death occurred due to severe ischemic injury to the central nervous system and limitations for further treatment.

## 4. Discussion

We conducted the first randomized pilot trial investigating the effect of cangrelor as an adjunct to standard antiplatelet therapy with P2Y_12_ receptor inhibitors in comatose patients following OHCA and PCI, treated with therapeutic hypothermia. We observed that an intravenous bolus and a four-hour infusion of cangrelor promptly and effectively suppressed platelet activity, thus addressing inadequate platelet inhibition until the onset of ticagrelor’s effect administered via enteral tube. Similar findings were previously reported in STEMI patients without OHCA when bridging inadequate platelet inhibition with a shorter two-hour cangrelor infusion [23]. Given the relevance of this topic to critical care, we believe that, in addition to late-breaking data being published, a comprehensive presentation of the full dataset from our study would be of additional value [24].

Baseline PRU values indicated HPR in the majority (77%) of patients, consistent with platelet reactivity data on mild therapeutic hypothermia [14]. In contrast to VerifyNow^®^ measurements, Multiplate^®^ ADP values were lower than expected, with basal HPR observed in only 30% of patients. A possible explanation for the difference is the methodology, as VerifyNow^®^ is a bedside test, whereas blood samples for Multiplate^®^ ADP were analyzed in the laboratory, leading to delayed sample processing due to transportation. The longer time to blood analysis is a likely cause of the lower AU values, as differences can arise as soon as one hour after sample collection [29]. Despite this, the effect of cangrelor was still evident in platelet inhibition measurements using both methods at one and three hours after the start of the infusion, consistent with findings from the CANTIC study, where adequate platelet inhibition with cangrelor was observed within five minutes of infusion initiation [23]. One and three hours after the end of infusion, i.e., five and eight hours after PCI, platelet inhibition did not differ between the groups in our study, indicating no interaction between ticagrelor and cangrelor. Similar findings were reported in patients with STEMI without OHCA, where platelet inhibition was monitored for two hours after the completion of cangrelor infusion [23]. In contrast to ticagrelor, an interaction between cangrelor and clopidogrel or prasugrel is described in the literature, necessitating delayed administration of the drug via an enteral tube until the end of cangrelor infusion [30,31]. This could further prolong the period of inadequate platelet inhibition. Findings from the FABOLUS-FASTER study in patients without OHCA confirmed reduced platelet inhibition with prasugrel application after a two-hour cangrelor infusion, with inadequate inhibition even more than 4–6 h after treatment initiation [32].

Differences in PRU and AU values between the groups at one and three hours after PCI also reflected a statistically significant difference in the proportion of patients with HPR. Three hours after PCI, HPR was still observed in half of the patients in the control group using the VerifyNow^®^ method. In a smaller study, HPR was reported after four hours in nearly a quarter of OHCA patients with the use of a one-hour cangrelor infusion, suggesting that the duration of infusion might have been too short [33]. Platelet inhibition measurements in our study indicated that a four-hour cangrelor infusion was adequate to bridge the period until the oral P2Y_12_ inhibitors became effective, even after eight hours post PCI. High platelet reactivity at five or eight hours was confirmed in only one patient in the cangrelor group, who already had the highest PRU values among all patients during cangrelor infusion (135 PRU at one hour and 125 PRU at three hours after PCI). While resistance to cangrelor has yet to be documented in the literature, it remains a possibility. Likewise, resistance to ticagrelor may contribute to HPR, as observed in patients with acute coronary syndrome without OHCA, with peak resistance occurring within the initial 24 h of drug administration [34]. Notably, there is a lack of literature data regarding ticagrelor resistance in OHCA patients.

Concurrently with our study, the ST-OHCA study was conducted at our department, where all OHCA patients undergoing PCI underwent follow-up coronary angiography or autopsy with sectioning of the coronary arteries in stented segments [13]. Among the total of 21 patients enrolled in both studies, two definite cases of ST were confirmed in the cangrelor group and three in the control group. The incidence of ST was inadequate for statistical analysis between groups. In 57% of the temporally defined confirmed ST cases in the ST-OHCA study, thrombosis occurred within the first day after PCI. This early occurrence of ST is consistent with results from a larger study, CHAMPION PHOENIX, which included patients with stable coronary artery disease or ACS without OHCA. In that study, most ischemic events were observed within the initial two hours following PCI [35]. The use of cangrelor in this study significantly reduced the number of ischemic events without an increase in bleeding complications [35]. In our study, despite cangrelor infusion and low measured platelet reactivity, ST occurred in two patients in the cangrelor group. This implied that clinical outcomes, including ST, are influenced by various factors beyond platelet inhibition. These factors may include patient-specific baseline characteristics, procedural angiographic nuances, and individual responses to treatment, some of which may currently remain unidentified. While our study shows immediate laboratory effects of antiplatelet therapy, it underscores the necessity for larger-scale studies to understand the real impact on clinical outcomes. Moreover, it is noteworthy to consider transient changes in laboratory platelet inhibition values following the cessation of cangrelor infusion. Larger studies may provide further insights into whether these changes correlate with adverse thrombotic events.

Due to the increased risk of bleeding associated with dual antiplatelet therapy in OHCA patients, we decided to exclude patients older than 70 years from the study design [6,36,37]. We did not observe a higher incidence of bleeding in the intervention group. Significantly more favorable pharmacokinetics of cangrelor offer an advantage over GPI, as its effect diminishes within a few minutes after discontinuation of the drug. In recent years, guidelines have recommended the use of GPI in OHCA patients only as rescue therapy in cases of poor PCI outcomes, as they are associated with more frequent significant bleeding events compared to cangrelor [18,19,38].

We did not observe significant differences in demographic data, circumstances of cardiac arrest, resuscitation, or angiographic characteristics between groups. The only statistically significant difference was the prevalence of multivessel coronary artery disease, which was present in three-quarters in the cangrelor group and in one-quarter of cases in the control group. However, PCI on non-culprit vessels was performed in only two patients in the cangrelor group, consistent with guidelines recommending treatment of only the culprit coronary artery during urgent PCI [6]. We found no disparities in survival rates, with favorable neurological outcomes between the groups. Although the sample is small, this is probably due to the primary cause of death in the majority of patients not being cardiogenic shock but rather severe ischemic injury of the central nervous system, leading to limitations in treatment options [6].

### Study Limitations

This pilot study has several limitations. Although it was randomized, patients in the control group did not receive a placebo. Because of the intervention with cangrelor and the associated costs, we recruited a relatively small number of patients, large enough for testing the primary endpoint hypothesis. This resulted in insufficient statistical power to detect differences in certain secondary clinical endpoints, such as the incidence of ST, survival, and neurological outcomes. A significant number of consecutive patients after OHCA and PCI were excluded after screening, most due to advanced age (n = 30), absence of ROSC (10), or logistical issues (12) primarily attributable to the COVID-19 epidemic. The findings of the study cannot be extrapolated to the group of OHCA patients treated with VA-ECMO, where there is an additional influence of extracorporeal circulation on blood coagulation. Another noteworthy limitation of the study is the low baseline levels of platelet reactivity measured using the Multiplate^®^ method, although this did not affect the outcomes related to the hypothesis.

Given the absence of significant bleeding in our study, it would be reasonable to consider using cangrelor bridging therapy in older patients in the future. Due to the effects of therapeutic hypothermia on platelet aggregation, the period of inadequate platelet inhibition during normothermia may be shorter, and a shorter infusion of cangrelor may be appropriate. Further research in this area is needed to answer the questions raised.

Despite the aforementioned limitations of the study, we believe that the results represent a significant contribution to the management of OHCA patients. Larger-scale studies are warranted to determine whether improved platelet inhibition will translate into clinical outcomes, potentially leading to a reduction in the incidence of ST among patients with adequate platelet inhibition.

## 5. Conclusions

In comatose survivors of OHCA undergoing PCI and TTM, cangrelor safely induced immediate and profound platelet inhibition, thereby bridging the “P2Y_12_ inhibition gap” after ticagrelor. There was no significant rebound in platelet reactivity after discontinuation of cangrelor infusion, indicating the lack of significant drug–drug interaction.

## Figures and Tables

**Table 1:** Demographic data and known risk factors for ischemic heart disease; circumstances of cardiac arrest and resuscitation of patients included in the study. BMI—body mass index; PCI—percutaneous coronary intervention; CABG—coronary artery bypass grafting; BLS—basic life support; EMS—emergency medical service; ROSC—return of spontaneous circulation; STEMI—ST elevation myocardial infarction; ECG—electrocardiogram.

	Cangrelor Group (n = 15)	Control Group (n = 15)	*p*-Value
Male gender	14 (93%)	15 (100%)	1.000
Age, years	56 ± 7	58 ± 6	0.317
BMI, kg/m^2^	27 ± 4	28 ± 7	0.547
Arterial hypertension	6 (40%)	5 (33%)	0.705
Hyperlipidemia	4 (27%)	3 (20%)	1.000
Diabetes mellitus	3 (20%)	2 (13%)	1.000
Smoking	7 (47%)	5 (33%)	0.456
Known ischemic heart disease	2 (13%)	1 (7%)	1.000
Previous myocardial infarction	0 (0%)	1 (7%)	1.000
Previous PCI/CABG	1 (7%)	1 (7%)	1.000
Witnessed cardiac arrest	14 (93%)	12 (80%)	0.598
Lay BLS	14 (93%)	13 (87%)	1.000
Time to EMS arrival, min	10 ± 6	8 ± 4	0.410
Shockable rhythm on arrival	14 (100%)	15 (100%)	NA
Time from EMS arrival to ROSC, min	12 ± 14	14 ± 8	0.522
Total downtime, min	21 ± 15	23 ± 9	0.758
STEMI on 12-lead ECG	9 (60%)	12 (80%)	0.427

**Table 2:** Admission laboratory findings and peak troponin levels during hospitalization in included patients. GFR—glomerular filtration rate; LVEF—left ventricular ejection fraction.

	Cangrelor Group (n = 15)	Control Group (n = 15)	*p*-Value
Arterial lactate, mmol/L	3.7 ± 2.3	4.8 ± 4.4	0.404
Arterial pH	7.27 ± 0.08	7.20 ± 0.11	0.072
Serum glucose, mmol/L	11.1 ± 4.2	13.3 ± 3.9	0.149
Creatinine, µmol/L	104 ± 15	104 ± 21	0.907
GFR < 60 mL/min	3 (20%)	5 (33%)	0.682
Hemoglobin, g/L	148 ± 12	145 ± 15	0.554
Platelet count, ×10^9^/L	243 ± 84	223 ± 66	0.484
Troponin I on admission, ng/L	3.424 ± 3.349	14.233 ± 24.884	0.107
Peak troponin I value, ng/L	77.179 ± 164.499	95.018 ± 112.963	0.732
LVEF < 40% on admission	5 (36%)	3 (23%)	0.678

**Table 3:** Angiographic characteristics of coronary angiography in enrolled patients. LAD—left anterior descending artery; LCX—left circumflex artery; RCA—right coronary artery; TIMI—thrombolysis in myocardial infarction; PCI—percutaneous coronary intervention; * *p* < 0.05.

	Cangrelor Group (n = 15)	Control Group (n = 15)	*p*-Value
Multivessel coronary disease	11 (73%)	4 (27%)	0.011 *
Acute lesion			
Left main	1 (7%)	0 (0%)	1.000
LAD	7 (47%)	10 (67%)	0.269
LCX/Ramus	3 (20%)	2 (13%)	1.000
RCA	3 (20%)	3 (20%)	1.000
TIMI flow 0–1 in acute lesion	10 (71%)	12 (80%)	0.682
PCI characteristics			
Lesions treated per patient	1.2 ± 0.7	1.0 ± 0	0.279
Number of stents per patient	1.5 ± 1.1	1.3 ± 1.2	0.753
Mean stent diameter, mm	2.5 ± 1.1	2.8 ± 0.8	0.387
Mean length of stented segments, mm	34.8 ± 19.0	29.2 ± 24.7	0.521
Successful PCI	13 (87%)	15 (100%)	0.483
Multivessel PCI	2 (13%)	0 (0%)	0.483
TIMI flow 2–3 at the end of PCI	13 (100%)	15 (100%)	1.000
Start of PCI to ticagrelor interval, min	56 ± 19	64 ± 19	0.252

**Table 4:** The proportion of patients with high platelet reactivity (HPR) assessed by the VerifyNow^®^ test. Baseline status before PCI and platelet reactivity at 1, 3, 5, and 8 h post-PCI are shown. PCI—percutaneous coronary intervention; * *p* < 0.05.

	Cangrelor Group (n = 15)	Control Group (n = 15)	*p*-Value
Baseline VerifyNow^®^ HPR	12 (80%)	11 (73%)	1.000
1 h	0 (0%)	10 (67%)	0.001 *
3 h	0 (0%)	7 (47%)	0.007 *
5 h	1 (8%)	4 (27%)	0.342
8 h	1 (20%)	2 (25%)	1.000

**Table 5:** The proportion of patients with high platelet reactivity (HPR) assessed by the Multiplate^®^ ADP test. Baseline status before PCI and platelet reactivity at 1, 3, 5, and 8 h post-PCI are shown. PCI—percutaneous coronary intervention; * *p* < 0.05.

	Cangrelor Group (n = 15)	Control Group (n = 15)	*p*-Value
Baseline Multiplate^®^ HPR	5 (33%)	4 (29%)	1.000
1 h	0 (0%)	5 (39%)	0.013 *
3 h	0 (0%)	5 (33%)	0.044 *
5 h	1 (8%)	2 (13%)	1.000
8 h	1 (20%)	1 (17%)	1.000

**Table 6:** Safety endpoint (bleeding events) and secondary clinical endpoints. BARC—Bleeding Academic Research Consortium; TIMI—thrombolysis in myocardial infarction; PCI—percutaneous coronary intervention; CPC—Cerebral Performance Category.

	Cangrelor Group (n = 15)	Control Group (n = 15)	*p*-Value
Bleeding events	3 (20%)	1 (7%)	0.589
BARC 2/3/5 or need for cangrelor discontinuation			
TIMI flow 2–3 at the end of PCI	13 (100%)	15 (100%)	1.000
Stent thrombosis	2 (14%)	3 (20%)	1.000
Survival to hospital discharge	10 (67%)	9 (60%)	1.000
Survival with CPC 1/2	10 (67%)	9 (60%)	1.000

## Data Availability

The data presented in this study are available on request from the corresponding author.
